# Design of an aircraft generator with radial force control

**DOI:** 10.12688/openreseurope.14684.1

**Published:** 2022-06-06

**Authors:** Christian Brecher, Stephan Neus, Marcus Gärtner, Hans-Martin Eckel, Maik Hoppert, Barry James, Chris Gerada, Michele Degano, Mohammad Reza Ilkhani, Mauro Di Nardo

**Affiliations:** 1 Laboratory for Machine Tools and Production Engineering (WZL), RWTH Aachen University, Campus-Boulevard 30, 52074 Aachen, Germany; 2 Romax Technology Ltd., a Hexagon company, Ergo House, Mere Way, Ruddington Fields Business Park, Nottingham, NG11 6JS, UK; 3 MSC Software GmbH, a Hexagon company, Am Moosfeld 13, Munich, 81829, Germany; 4 Power Electronics, Machines and Control (PEMC) Research Centre, University of Nottingham, Engineering Department, Jubilee Campus, Wollaton Road, Nottingham, NG8 1BB, UK

**Keywords:** Aircraft generator, radial force control, bearing friction, bearing loads, efficiency, validation, test rig, shaft-bearing system

## Abstract

With the increasing electrical energy demands in aviation propulsion systems, the increase in the onboard generators’ power density is inevitable. During the flight, forces coming from the gearbox or gyroscopic forces generated by flight manoeuvres like take-off and landing can act on the generators' bearings, which can lead to wear and fatigue in the bearings. Utilizing the radial force control concept in the electrical machine can relieve loads from the bearings that not only minimize the bearing losses but also increase bearing life. The objective of the MAGLEV project (Measurement and Analysis of Generator bearing Loads and Efficiency with Validation) is to study, demonstrate, and test a new class of high-speed generators with radial force control. In this paper, design steps of this type of generator and its test rig are presented and the measurement methodology used for radial force control is explained. The concept is developed in an electrical machine and is validated on a test rig by measuring required parameters like shaft displacement, vibrations and bearing temperature. Additionally, the friction moment of each generator’s bearings is measured and validated in a separate test rig under comparable conditions to the bearing loads in the generator. Therefore, a novel approach to determine precisely the bearing friction in a radial load unit, rotatably supported by an additional needle bearing is used, which shows a good agreement with the calculated friction. Furthermore, new calculation methods for the operating behavior of cylindrical roller bearings with clearance are presented, which are utilized in the generator test rig.

## 1 Introduction

**Figure 1.**
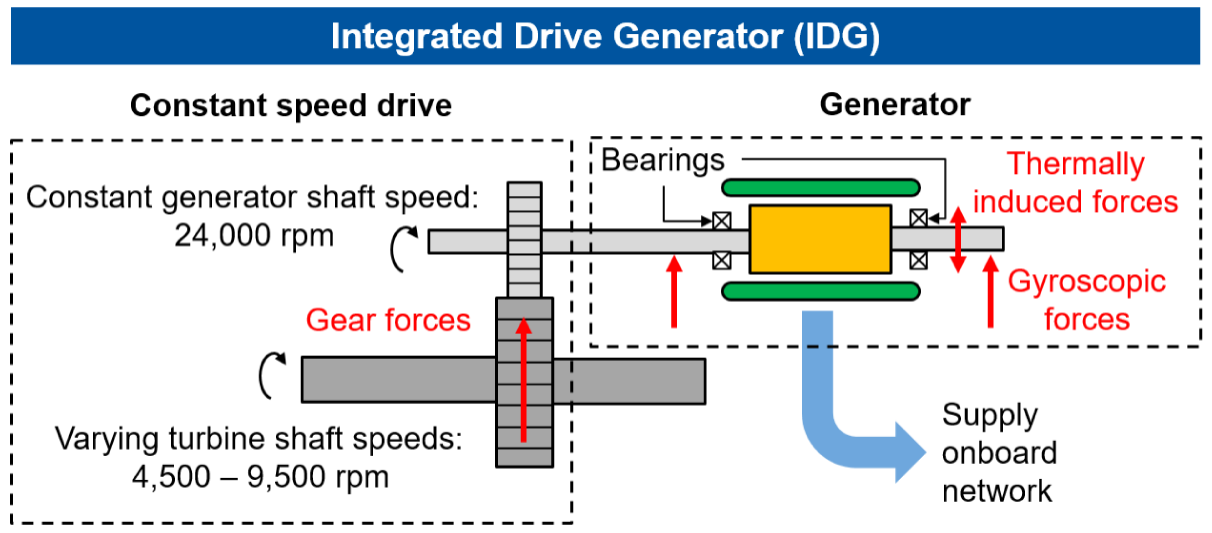
Schematic structure of the IDG (integrated drive generator).

Due to increasing passenger numbers in the civil aviation sector, there is a trend towards larger long-haul aircraft, such as the Airbus A380 with up to 853 passengers ^1^, which needs more electrical energy and increases the demand for efficient and compact generators. This requirement is analyzed by Madonna, who has studied the development of aircraft generators in the 20th and 21st centuries and who has estimated an energy density of 40 kVA/kg for electrical machines in aircraft by the year 2050 ^2^. Additionally, Madonna presents a highly efficient and compact permanent magnet (PM) generator with an energy density of 16 kVA/kg, which can meet future requirements. Electrical on-board generators such as the integrated drive generator (IDG), converts the varying speeds of the turbine shaft to a constant speed of the generator shaft by a hydraulic speed converter (Constant Speed Drive, CSD). The schematics of IDG that feeds the onboard network is shown in Figure 1. In this example, the IDG is used for aircraft with a high power demand at a constant frequency. Varying rotational speeds between 4,500 rpm and 9,200 rpm of the turbine shaft are converted to a constant maximum speed of 24,000 rpm by a Constant Speed Variable Transmission system (CVT). However, in this concept, the radial interaction forces can be transmitted to the generator shaft by misalignment of the gear shafts and machine vibrations ^3^.

Due to the high speeds and generated heat during the operation IDGs, they have a cooling system. If the cooling system fails, thermally induced interference forces can act on the generator and consequently on the bearings, which can lead to a bearing failure ^4^. In addition, during the take-off and landing manoeuvres of the aircraft, gyroscopic forces can act on the generator’s shaft. However, as the onboard generators are extremely important components for the aircraft and its power supply chain, they must be protected from mechanical overload. One solution to minimise these interference forces is the radial force control (RFC) concept studied in ^5^. As the first approach, this technology was tested in a six-pole permanent magnet machine with a maximum power of 150 kW, and thermal and dynamical design was introduced in ^6^. To minimize winding losses, the stator, which is spatially separated from the rotor, has been cooled by a constant oil flow. In this machine, the radial force control has been used to suppress vibrations caused by imbalances for speeds up to 3,000 rpm ^5^. Using displacement signals, the eccentricity position of the shaft has been determined and compensated by radial forces induced in the generator. The radial force control technology has been used for starter engines of aircraft, too, instead of pneumatic power needed in auxiliary systems ^7^. The objective of the MAGLEV (Measurement and Analysis of Generator bearing Loads and Efficiency with Validation) project is to develop and upgrade the control of the radial force control generator for speeds up to 20,000 rpm and greater loading conditions. Radial force control aims to relieve the interference forces acting during the operation of the bearings and to reduce the bearings’ friction losses. The radial force control for bearing load relief will be tested and validated on a newly developed generator test rig, which not only emulates the radial interference forces of the constant speed drive but also the forces applied during start and landing manoeuvres. Therefore, mechanical and electrical engineering scientists from RWTH Aachen University, the University of Nottingham and the company Hexagon are working together to meet the challenges of developing and testing this new technology. In the following, the methodology and procedure in this project are outlined. However, the methods developed, such as the measurement of bearing friction under radial load, can be transferred to comparable scientific investigations.

## 2 Design of the generator test rig

### 2.1 Overview

**Figure 2.**
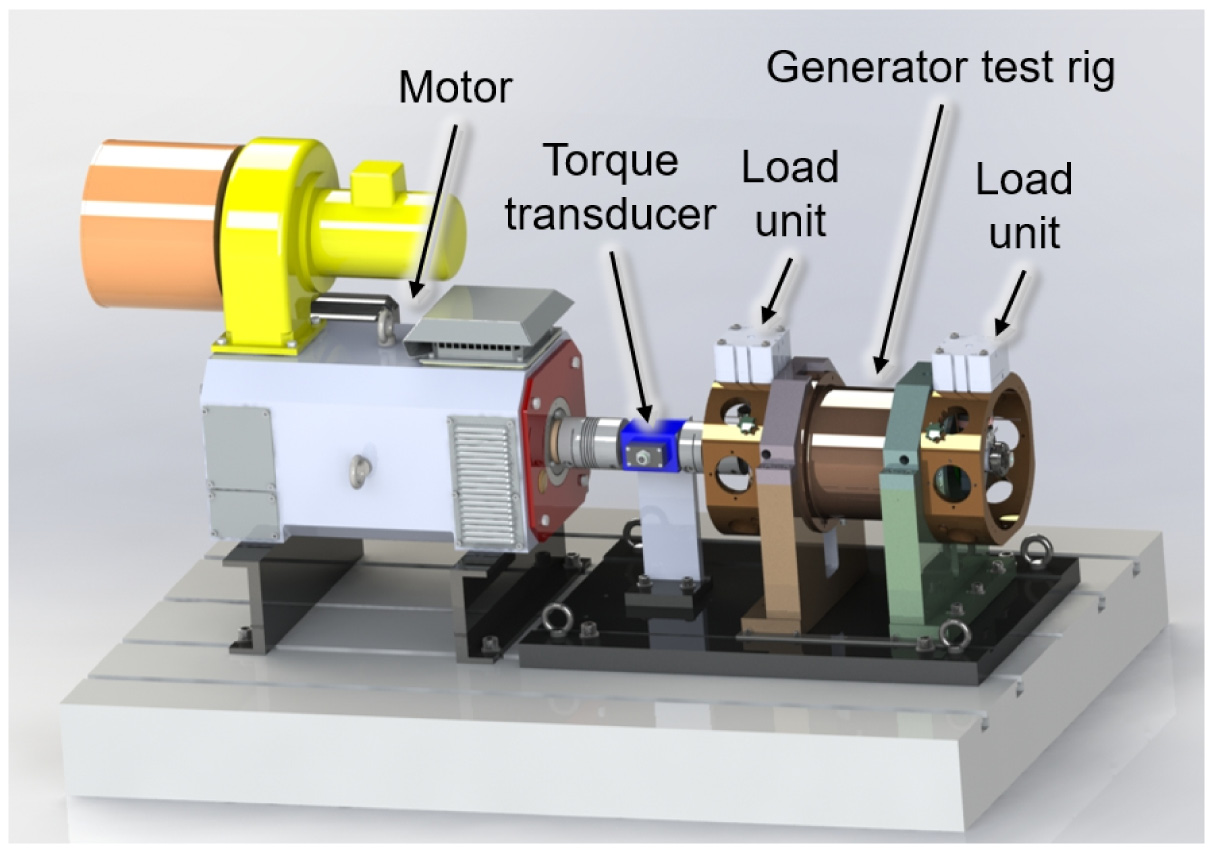
Generator test rig.

To test the radial force control for bearing relief a test rig is designed that can create comparable loading to the operational conditions of the aircraft, as shown in Figure 2. The generator is driven by a synchronous motor with a maximum speed of 20,000 rpm. The torque transferred over the total drive train is measured by a torque transducer. Two load units are considered on both ends of the generator’s shaft, that can emulate different loading conditions including gyroscopic moments interference forces seen during real operation. A mechanism is considered between the load units and generator housing to move the load units circumferentially and apply force to the generator shaft from different angular positions with respect to the rotation axis. Using the measured radial shaft displacement, the bearing loads can be determined indirectly based on load-displacement ratios ^8^. By decoupling the machine from the motor, the generator can be used in motor state and the radial force control can be tested in a further application.

### 2.2 Electrical part and sensors

**Figure 3.**
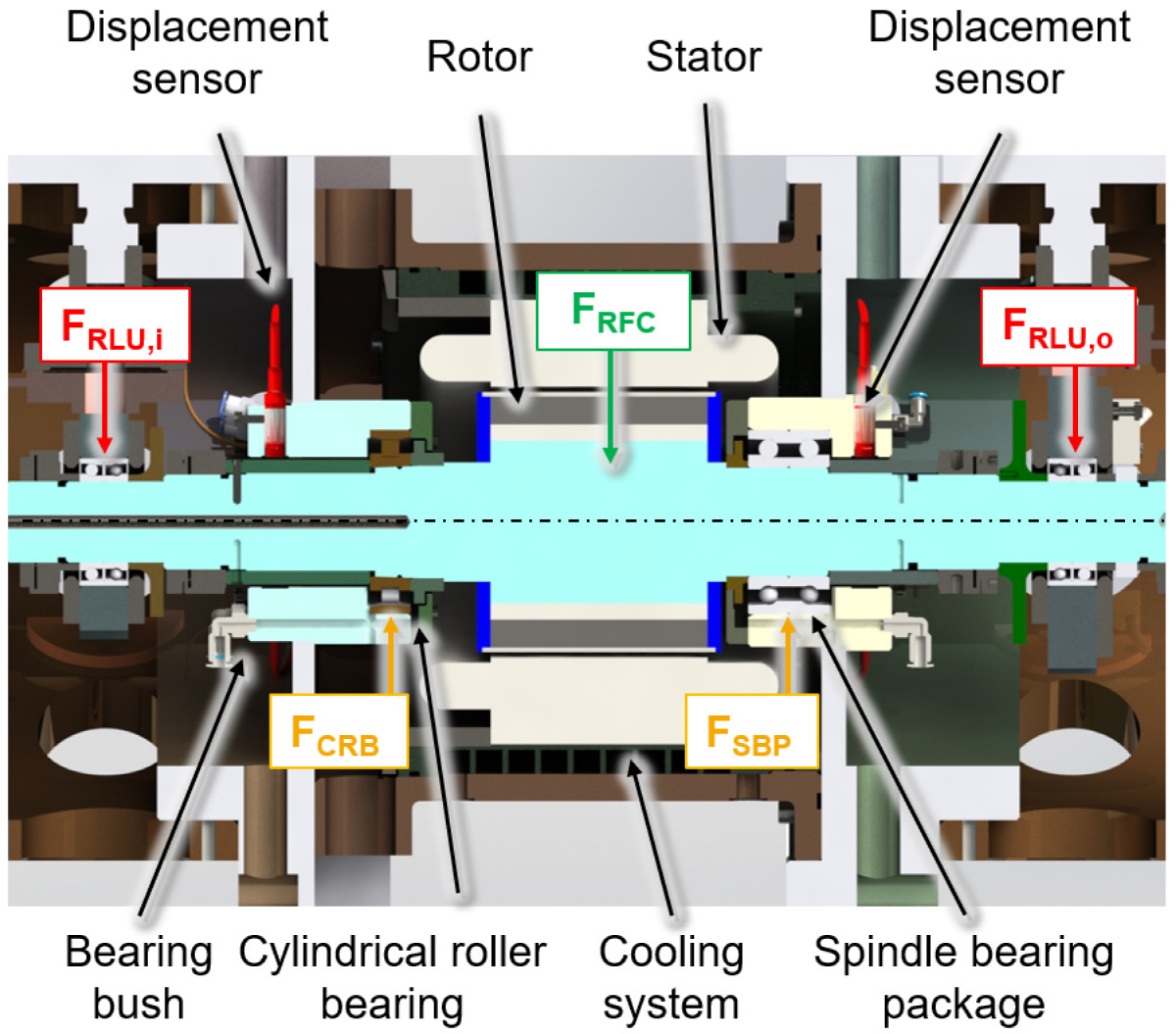
Cross section of the generator test rig. RFC, radial force control; RLU, radial load unit; SBP, spindle bearing package; CRB, cylindrical roller bearing.

The electric generator part is shown in a cross-section view with the integrated sensors in Figure 3. The generator is designed in a fixed-floating arrangement, with a cylindrical roller bearing (CRB) with clearance on the driven side (input) and a preloaded spindle bearing package (SBP) on the non-driven side (output). The radial shaft displacement, which is the input value for the radial force control, is measured close to the two bearing positions using three non-contact eddy current sensors arranged at 120° to each other on both sides. The interference forces of the load units *F*_*RLU*, *i/o*_ are compensated by an electromagnetic force *F _RFC_* from the generator to reduce loads of the cylindrical roller bearing *F _CRB_* and the spindle bearing package *F _SBP_* .

**Figure 4.**
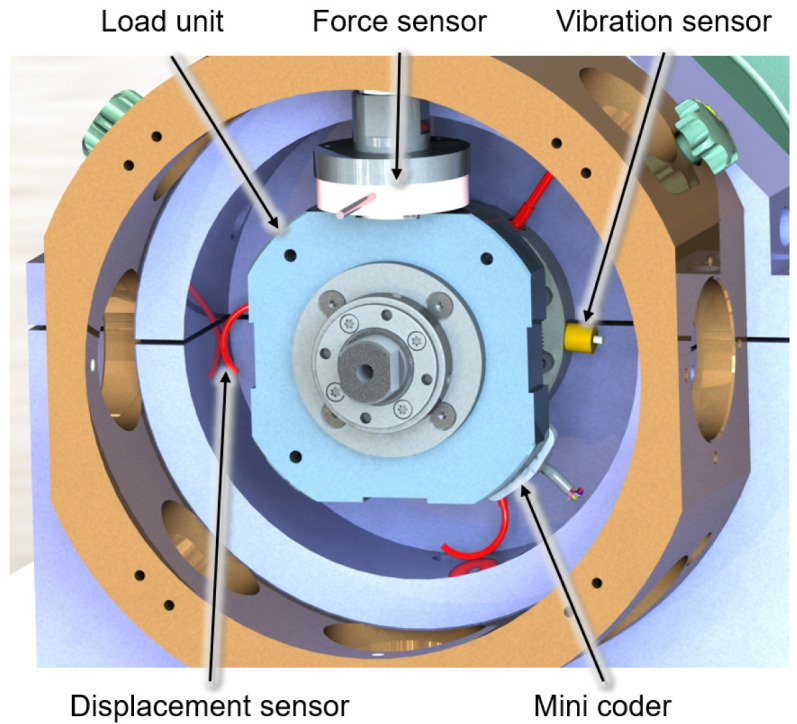
Load unit with sensors.

The compensating force is analytically determined based on force and torque equilibria so that loads of the generator bearings are minimized. The radial force control is validated using acceleration, temperature, rotational speed and torque signals. The sensor system to measure the operational behaviour of the bearing is part of the bearing monitoring system. Figure 4 shows the output side of the generator test rig with a load unit and some of the sensors. Two piezoelectric vibration sensors mounted on both sides of the bearing bushes provide information about the dynamic behaviour of the test rig and the lubrication condition in the bearing by high-frequency measurements of the acceleration level of up to 51.2 kHz. The electromagnetic encoder measures the speed and angular position of the generator shaft and is the control input for the RFC application. The force sensors measure the actual force applied by the load units and also provide feedback on pneumatic cylinders’ force control. Furthermore, the bearing temperatures are measured with resistance thermometers to determine the steady-state conditions. The cage speed of the generator bearing is monitored by infrared sensors to validate the slip and skidding behaviour of the cylindrical roller bearing in critical operational conditions ^9^. The measured torque on the drive train can also be a good variable for evaluating radial force control. However, the expected load torques are up to 120 Nm, which is significantly higher than the expected bearing friction losses, so the validation of the single bearing friction based only on the measured gross input torque is not sufficient. Therefore, further investigations on a single bearing test rig with a friction measurement system under comparable conditions to the generator test rig are necessary. So another test rig is considered for this purpose with its measurement set-up that is described in section 5.

## 3 Radial force control

The basic idea of radial force control is to detect interference forces applied from outside, in the case of the generator test rig by the load units, and to compensate them by a counteracting force in the electrical generator. This leads to bearing load relief resulting in reduced bearing friction, a higher generator performance and in the long term also to increased reliability and lifetime. In the past, this technology has already been successfully tested for the suppression of vibrations in a multi-sector permanent magnet machine ^5, 10^. The shaft displacement is the input signal for the radial force control, the corresponding counterforce is then applied to the shaft ^11^. Since the radial force applied by the generator cannot be measured directly, a ratio between the generator current and the actual force applied must be identified in preliminary investigations. The procedure for determining this ratio is described in the following section.

### 3.1 Calibration process

**Figure 5.**
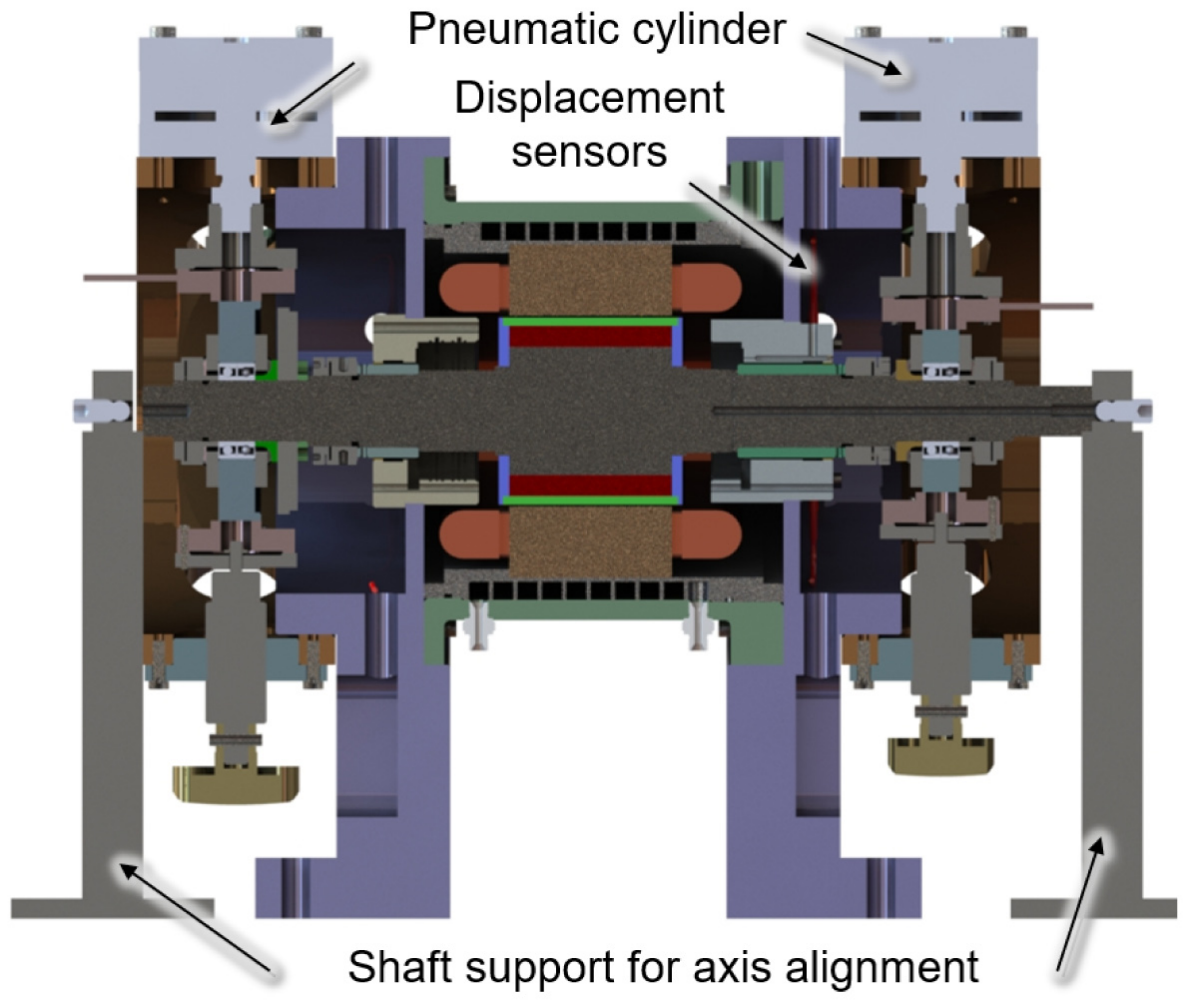
Cross section of the the calibration set up.

Direct measurement and calibration of the generator force can be achieved by removing the generator bearings and simply supporting the generator shaft on the load units. The test set-up for this calibration measurement is shown in a cross-sectional view in Figure 5. The load units with the pneumatic cylinders are removed in this setup. For rough axis alignment, the shaft is additionally supported by two shaft supports on both sides, which are omitted for fine adjustment. 

**Figure 6.**
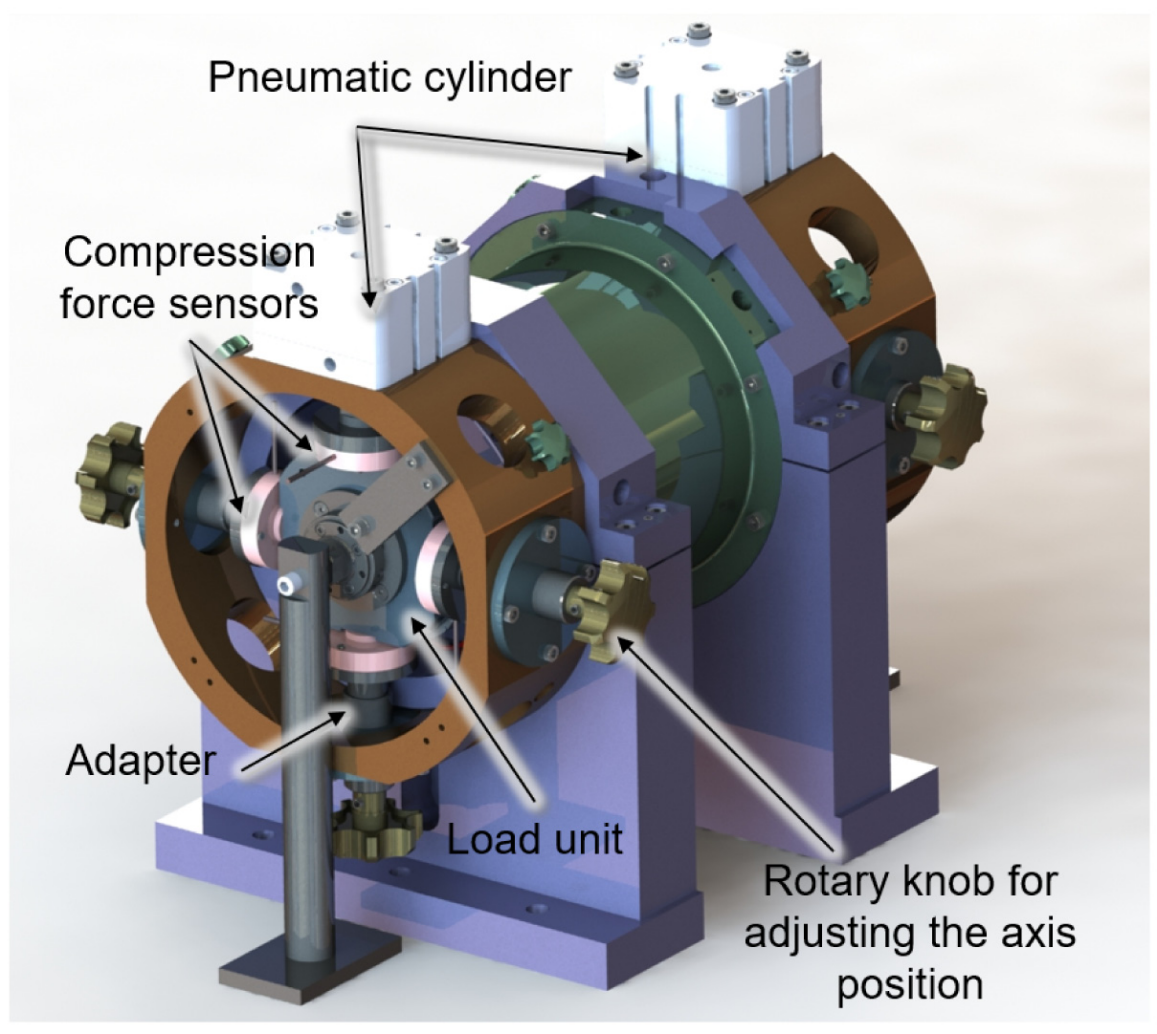
Set up of calibration measurement.

The load units on both sides are supported in four directions by compression force sensors, which are attached to the housing by adapters shown in Figure 6. The adapters can be adjusted in their axis position by a rotary knob so that the shaft can be aligned concentrically to the generator. For the fine alignment of the shaft, the displacement sensors of the bearing monitoring system can be used. If the radial force is now adjusted to the rotor by the generator current, the amount and direction of this force can be measured by the compression force sensors on the load units. The calibration process will be carried out for low speeds up to 5,000 rpm to avoid overloading of the load unit bearings due to imbalances. Due to the omission of the generator bearings, the radial force applied by the generator can cause a high deflection of the rotor. To ensure that no collision of rotor and stator occurs in the calibration process, the displacement behaviour of the shaft for different generator forces was simulated with the MATLAB-based program MTPlus, which was previously developed at the WZL ^12^ (see *Software availability*). The software is a combination of the the shaft calculation program NewSpilad ^13^ and single bearing calculation program Winlager ^14^ also developed at WZL. The calculation principles of the program are described, among other things, in the following sources ^15– 19^. For these and all following calculations the internal geometries of the bearings, such as osculation and roller profiling, which are manufacturer-specific and are not usually published, were measured on a coordinate measuring machine. A comparison of the MTPlus simulations with the commercially available shaft-bearing calculation software Mesys showed good agreement ^20^. An open source alternative for the calculation of rotordyanmic and shaft bending is the software ROSS ^21^. This Python-based program does not offer the full range of functions in comparison to commercially available calculation programmes, e.g. the bearings are just approximated as simple spring-mass dampers, but it can be used to calculate the shaft displacement or for the dynamic frequencies of the shaft-bearing system.

**Figure 7.**
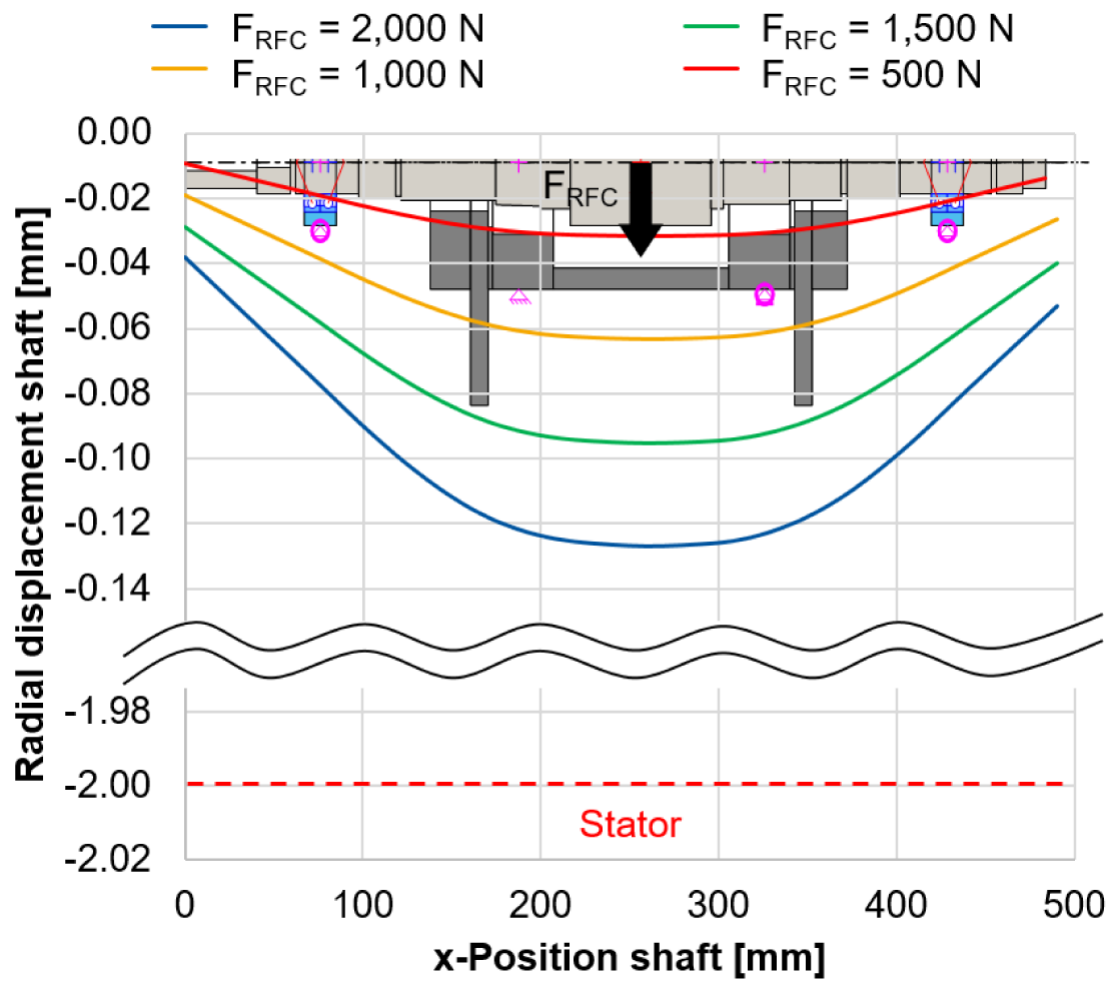
Radial shaft displacement (bearingless mode). RFC, radial force control.

In Figure 7, the radial shaft displacement is shown for varying radial forces of the electrical generator *F _RFC_* and a rotational speed of 5,000 rpm (see *Underlying data* ^22^).

The relatively low stiffness of the force sensors compared to the housing of approx. 13,000 N/mm, based on the sensor specifications, is considered to support the load units. The bearings of the load units (type 71906) are preloaded with 3 *µ*m in a rigid arrangement with a shaft interference fit of 2 *µ*m. Even for the highest radial force of 2,000 N, the radial displacement of the rotor of 0.129 mm is still below the air gap between the rotor and stator which is 2 mm. Therefore it is expected, that there will be no collision between rotor and stator during the calibration tests. This calibration method is a novel approach to directly measure the radial force applied by the generator including side effects. The methodology is validated during the commissioning phase and gives an accurate indication of the potential of radial force control.

### 3.2 Control loop strategy

**Figure 8.**
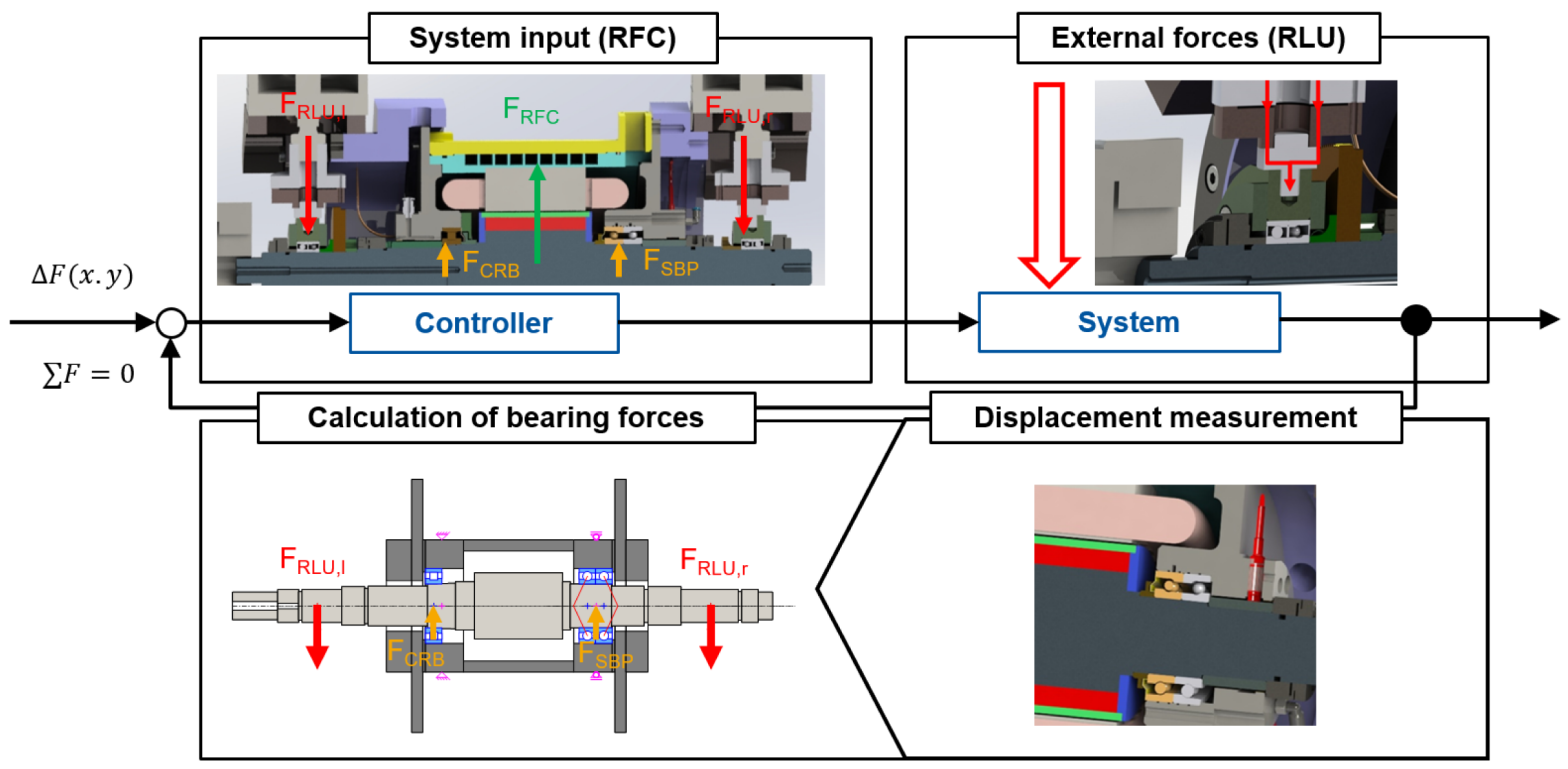
Simplified radial force control (RFC) loop. RLU, radial load unit; SBP, spindle bearing package; CRB, cylindrical roller bearing.

The design of the radial force control loop has a major influence on the performance of the bearing load relief. An efficient implementation of the algorithm to apply radial force can lead to reduced bearing friction, better power transmission and, in the long term, also to increased reliability and lifetime. In practice, the external loads or interference forces on the generator are not known and can only be determined indirectly by the shaft displacement. The simplified control loop for radial force control in the generator test rig is given in Figure 8. According to the application, the bearing relief should be implemented as follows. First, an external load is radially applied to the shaft by the load unit. The bearing forces can be determined by the shaft displacement with a shaft-bearing simulation model. In the next step, the radial force of the generator is determined so that the bearing loads are minimized. This leads to a lower shaft displacement. In addition, the bearing relief can be validated in extended runs by measuring the bearing temperature and power losses.

### 3.3 Increased performance due to bearing relief

**Figure 9.**
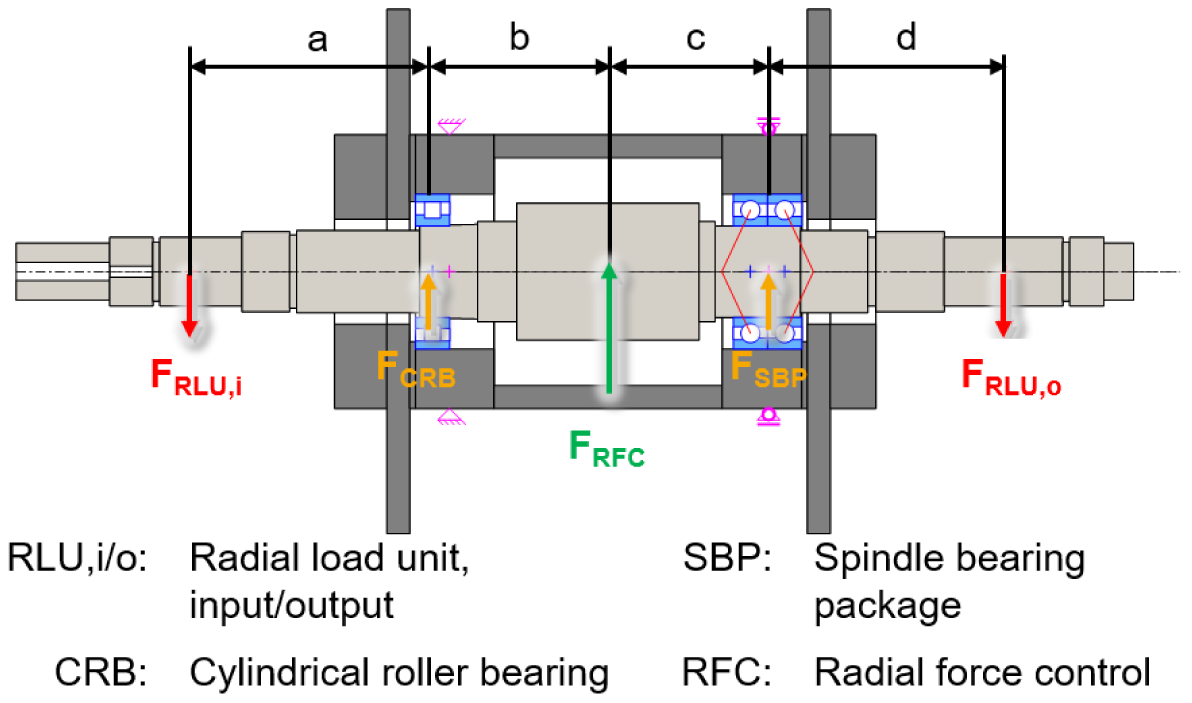
MTPlus model of the generator test rig.

To illustrate the effect of bearing relief by radial force control, the shaft bearing system of the generator test rig was simulated using the program MTPlus ^12^ (see *Software availability*). The methods necessary for the friction calculation approach for spindle bearings is set out in 17. The following calculations can also be performed using commercial bearing calculation software, e.g. Mesys. Currently, there is no open source single bearing calculation software available to determine, e.g., contact stresses, bearing friction or life. Gupta gives a good overview to this issue ^23^. However, the simulation model of the electrical generator is shown in Figure 9, and corresponding data is available in *Underlying data* ^22^. The cylindrical roller bearing is slightly radially preloaded, and the spindle bearing package is axially preloaded in an O- arrangement. The forces applied by the radial load units are adjusted so that the radial bearing forces of the cylindrical roller bearing and the spindle bearing package are equal. The load ratio between left and right radial load unit for uniform radial loading of the generator bearing is given in eq. 1 ^24^.


$$\;F_{RLU,i}=\frac{2\cdot a+b+c}{b+c+2\cdot d}\cdot F_{RLU,o}\;(1)$$

The ratio between the compensating force to be applied (radial force control RFC) and the interference forces of the radial load units results from the equilibrium of forces and moments. The bearing forces for the spindle bearing package *F _SBP_* and the cylindrical roller bearing *F _CRB_* in eq. 2 and eq. 3 have to be minimized.


$$F_{SBP}=\frac{F_{RLU,o}\cdot (a-(b+c+d))+F_{RFC}\cdot b}{b+c}\;(2)$$

$$F_{CRB}=\frac{F_{RLU,o}\cdot (d-(a+b+c))+F_{RFC}\cdot c}{b+c}\;(3)$$

**Figure 10.**
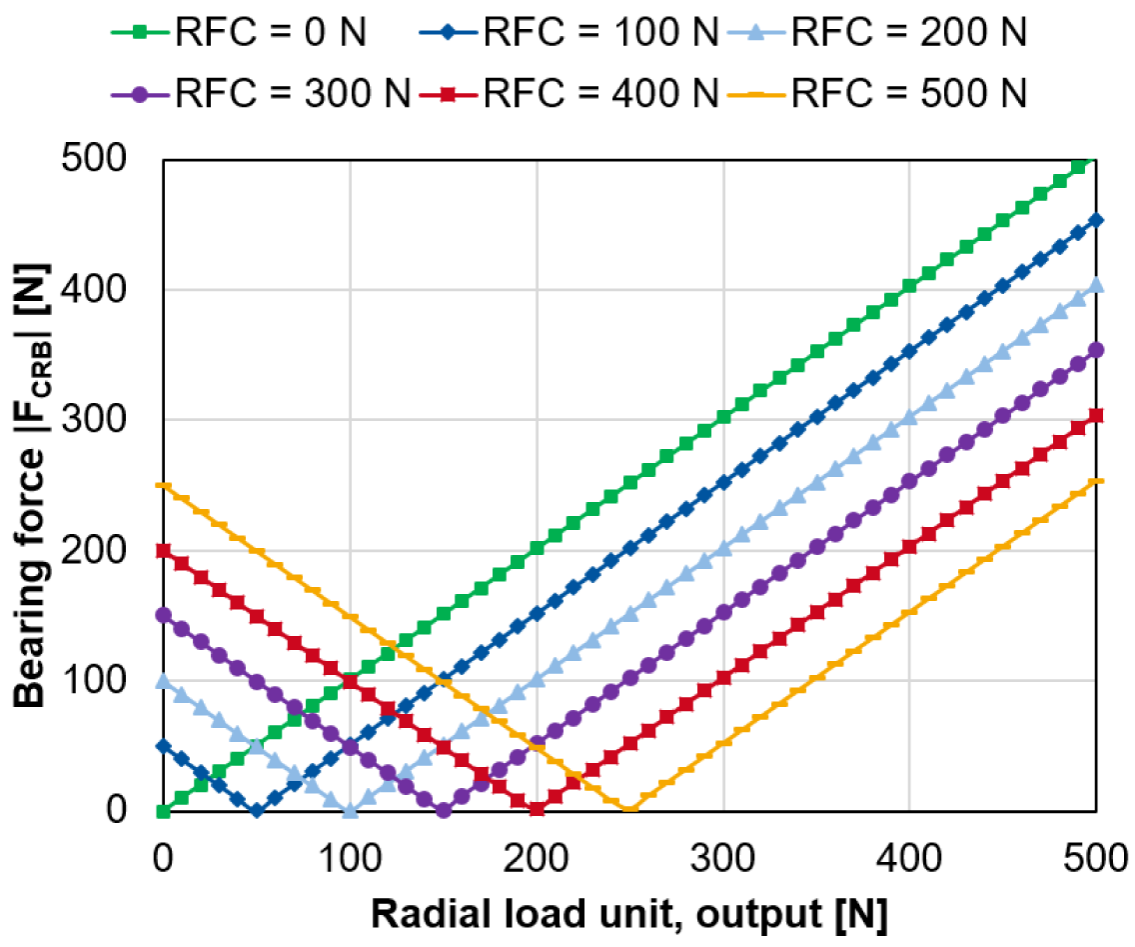
Variation of the generator radial force control (RFC). CRB, cylindrical roller bearing.

For an increasing radial force of the load unit on the output side, Figure 10 shows the magnitude of the bearing force of the cylindrical roller bearing for a varying compensation force of the radial force control *F _RFC_* (see *Underlying data* ^22^).

Based on the variation calculations, the following ratio is derived in eq. 4 between the necessary generator force *F _RFC_* to compensate for the load on the radial load units *F _RLU,i/o_.*


$$\;F_{RFC}=2\cdot F_{RLU,i/o}\;(4)$$

In the best case, the radial force control can fully compensate the interference forces applied by the radial load units on the bearings.

**Figure 11.**
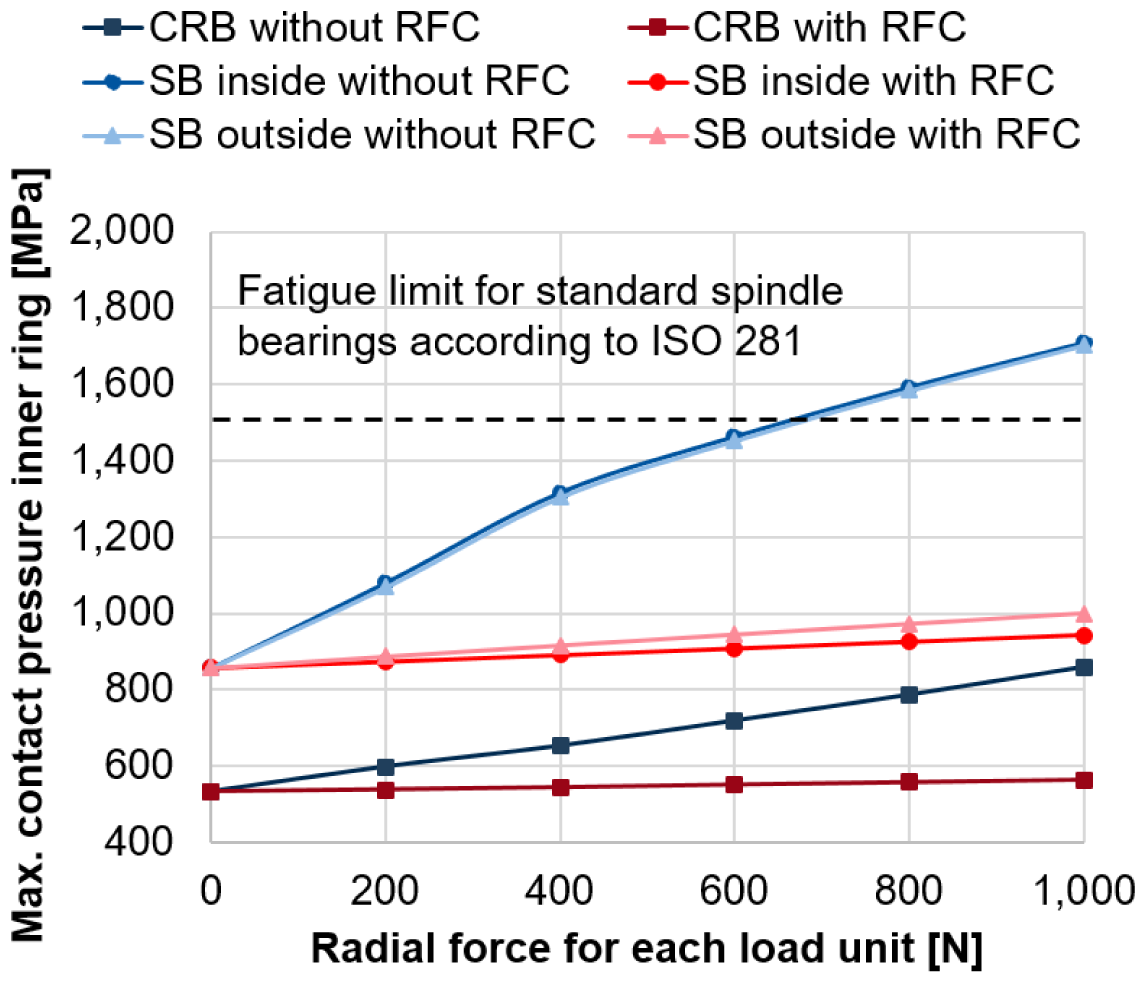
Maximum contact pressure on the inner ring. RFC, radial force control; CRB, cylindrical roller bearing; SB, spindle bearing.

Figure 11 shows the critical maximum contact pressures for the inner ring of the bearings of the generator for increasing forces of the two radial load units with and without radial force control for the maximum rotational speed of 20,000 rpm (see *Underlying data* ^22^). Without radial force control and load unit forces above 600 N, the critical contact pressure of 1,500 MPa is exceeded ^25^. With radial force control and increasing forces of load units, the maximum contact pressures of the inner ring are kept on a constant level below this critical value.

**Figure 12.**
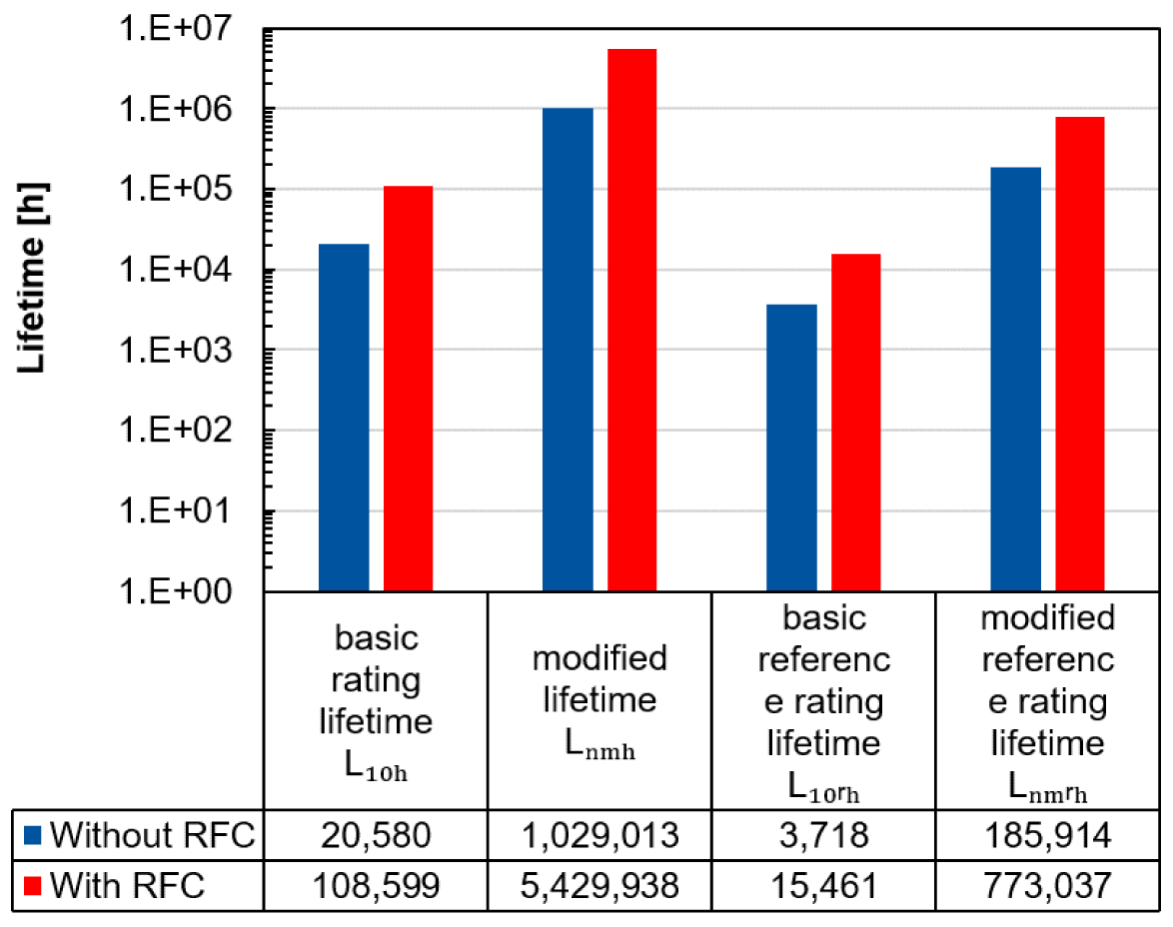
Lifetime calculation of the inside positioned spindle bearing with and without radial force control (RFC).

The advantage of radial force control can also be illustrated by calculating the lifetime. Figure 12 shows the lifetimes according to the standards ISO 281 and ISO/TS 16281 for the inside positioned spindle bearing by radial forces of 1,000 N per load unit and a rotational speed of 20,000 rpm with and without radial force control ^25, 26^ (see *Underlying data* ^22^). Generally, the ISO/TS 16281 standard calculates the internal load distribution in the bearing, which means that the bearing clearance and tilting can also be taken into account. Conversely, in ISO 281 the forces applied on the bearing are combined in an equivalent bearing load. In both standards the bearing specific value of the load carrying capacity *C _r_* is required. Furthermore, in both standards the lubricant influence *a _iso_* and the reliability *a*_1_ can be considered in the modified lifetime *L _nmh_* and the modified reference rating lifetime *L _nmrh_*. With the basic equivalent load rating *P _r_* and the exponent *p* (balls: 3, rollers: $\frac{10}{3}$ ), the modified lifetime *L _nmh_* can be calculated with eq. 5.


$$\;L_{nmh}=a_{1}\cdot a_{iso}\cdot L_{10h}=a_{1}\cdot a_{iso}\cdot {\left(\frac{C_{r}}{P_{r}}\right)}^{p}\;(5)$$

The lifetimes for the modified and modified reference rating lifetime are higher compared to the others, as it can be assumed that the lubricant film builds up sufficiently due to the high speeds and thus reduces wear. In all cases, this example shows that the bearing relief by radial force control leads to an increase in the predicted lifetime.

**Figure 13.**
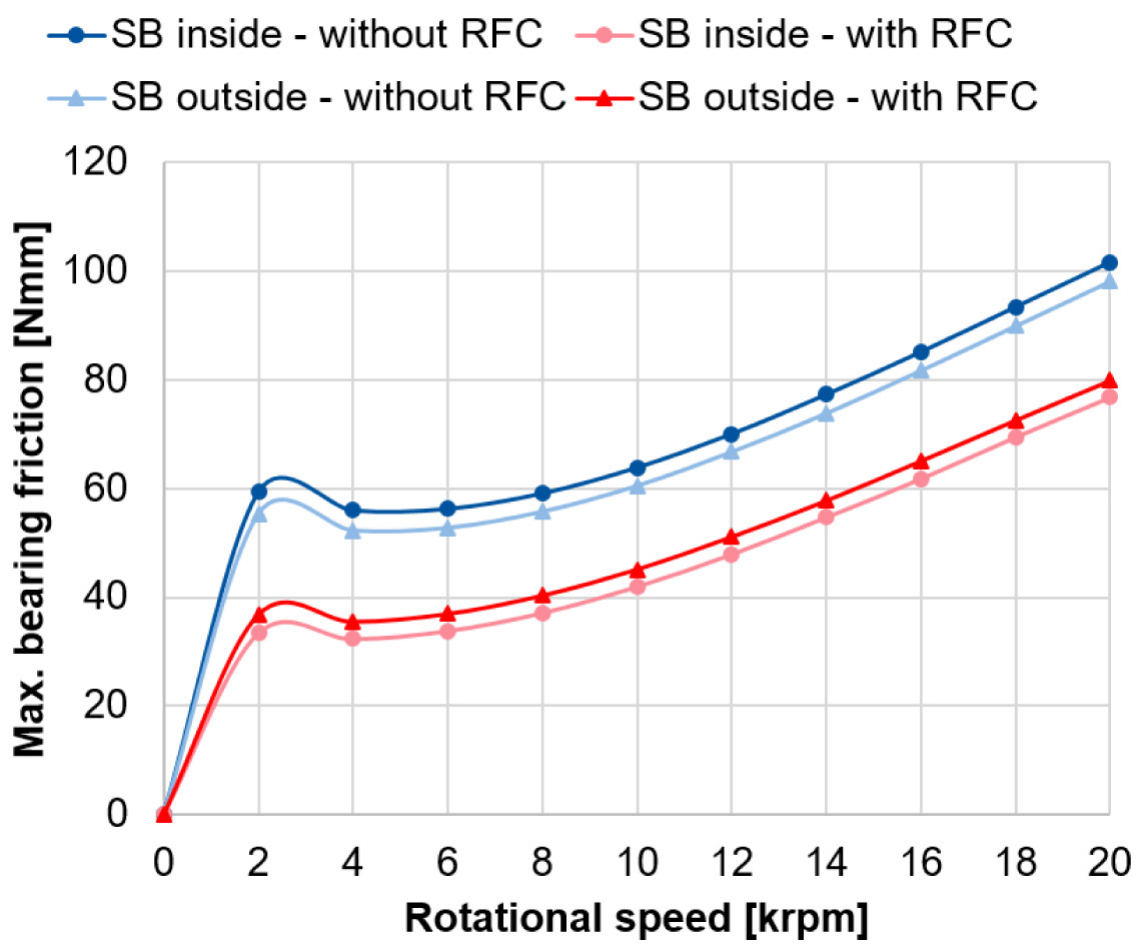
Maximum friction in the spindle bearings. RFC, radial force control; SB, spindle bearing.

The calculated bearing friction for the spindle bearing package with and without radial force control based on the approach of Rossaint ^17^ is shown in Figure 13 (see *Underlying data* ^22^). This holistic approach makes it possible to determine the bearing friction based on the relative speeds and the forces in rolling contact by using an experimentally determined transfer function ^27, 28^. In addition, this approach also considers the influence of cage friction, which has an impact on the operational behaviour high-speed rolling bearings. For the calculations the ball guidance method according to Tüllmann is used ^16^. With radial force control, the calculated bearing frictional torque is significantly lower due to the smaller contact pressures in the rolling contact, and thus, the lower friction. By using radial force control, the bearing friction can be reduced by around 33 %. In general, the calculated friction curves show the classical Stribeck curve behaviour, where a local friction minimum occurs at the transition from mixed to fluid friction at 4,000 rpm ^29^. The example calculations show that the bearing loads, the bearing friction and the lifetime of the spindle bearings can be significantly improved by means of radial force control. Similar results can also be achieved with cylindrical roller bearings, which will be the focus of the next section.

## 4 Cylindrical roller bearing with clearance

**Figure 14.**
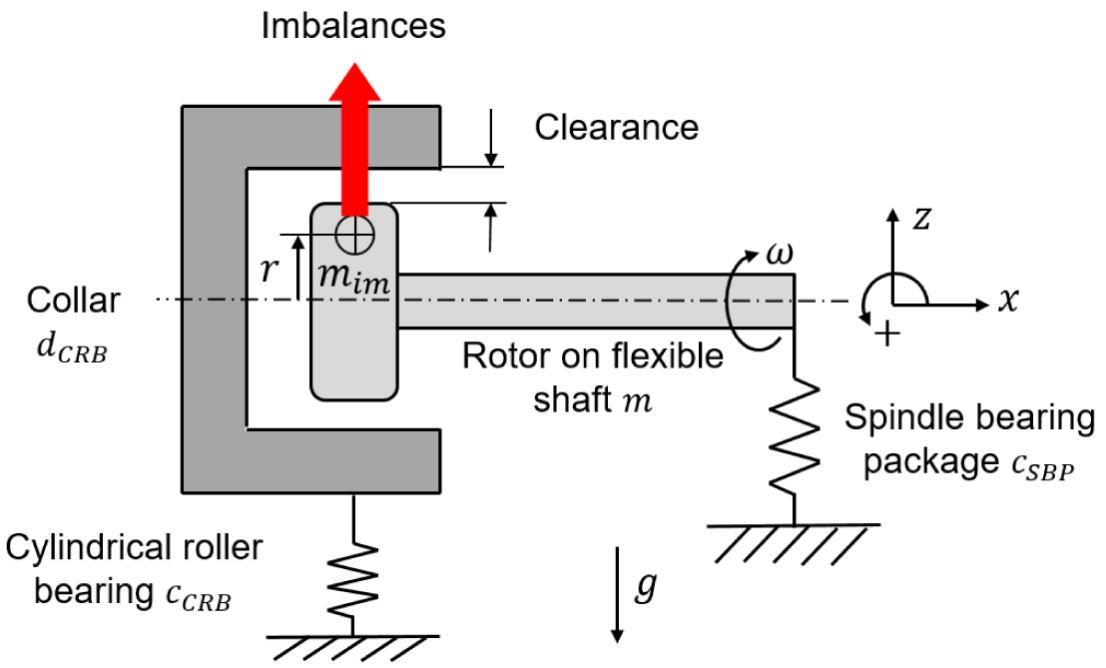
Shaft bearing system as spring mass damper.

Typically, cylindrical roller bearings in the high-speed application are operated with a light preload to ensure sufficient stiffness and to provide an even load distribution, so that wear in the rolling contact due to overload is minimised ^30^. However, the effect of radial force control can be increased by a small clearance in the bearing and the resulting reduced stiffness. Therefore, the following section focuses on the influence of radial clearance of the cylindrical roller bearing on the rotor dynamics and the resulting bearing losses. Particularly at high speeds in the generator, the non-linear stiffness behaviour of the cylindrical roller bearing for discrete operating points is changing in the time domain, which must be taken into account in the design. Since the simulation software Nexus also allows the transient calculation of cylindrical roller bearings, additional design calculations of the cylindrical roller bearing were carried out in the frequency and time domain. The software has been previously described in 31, 32 and 33. The following calculations are a new extension of the program Nexus, which, as far as is known, cannot be carried out by common commercial or open source software (see *Software availability*). The operating behavior of the cylindrical roller bearing of size N1008 used in the generator test rig was calculated for speeds up to 35 krpm without external loads. The underlying rolling contact geometries, such as the roller profiling, which are the bearing manufacturer’s special knowledge, were determined on a coordinate measuring machine and can therefore not be disclosed. As a basis for the following calculations, the shaft-bearing system of the generator with the cylindrical roller bearing with radial clearance can be modeled simplified as a spring-mass oscillator, which is shown in Figure 14. At standstill, the stiffness of the system is zero. With increasing speed, the bearing clearance is progressively reduced due to the imbalance of the shaft and the rollers.

The spindle bearing package is preloaded so that the system has a tilting stiffness and is a cantilevered system at low speeds. For the shaft-bearing system, the following simplified motion equation for the speed increase (run-up) with the total mass *m*, the rotational speed *ω*, the imbalance mass *m _im_*, the stiffness of the spindle bearing package *c _SBP_* and the stiffness of the cylindrical roller bearing *c _CRB_* can be established in eq. 6.


$$F_{run-up}=m\cdot \ddot{z}=m_{im}r\omega^{2}-mg+c_{SBP}\Delta z^{*}+c_{CRB}\Delta z^{**}\;(6)$$

In the case of a speed decrease (run-down), the cylindrical roller bearing with clearance responds like a damper *d _CRB_*, the equation eq. 7 of motion then changes as follows.


$$F_{run-down}=m\cdot \ddot{z}=m_{im}\;r\omega^{2}-mg+c_{SBP}\Delta z^{*}+d_{CRB}\Delta {\dot{z}}^{**}\;(7)$$

**Figure 15.**
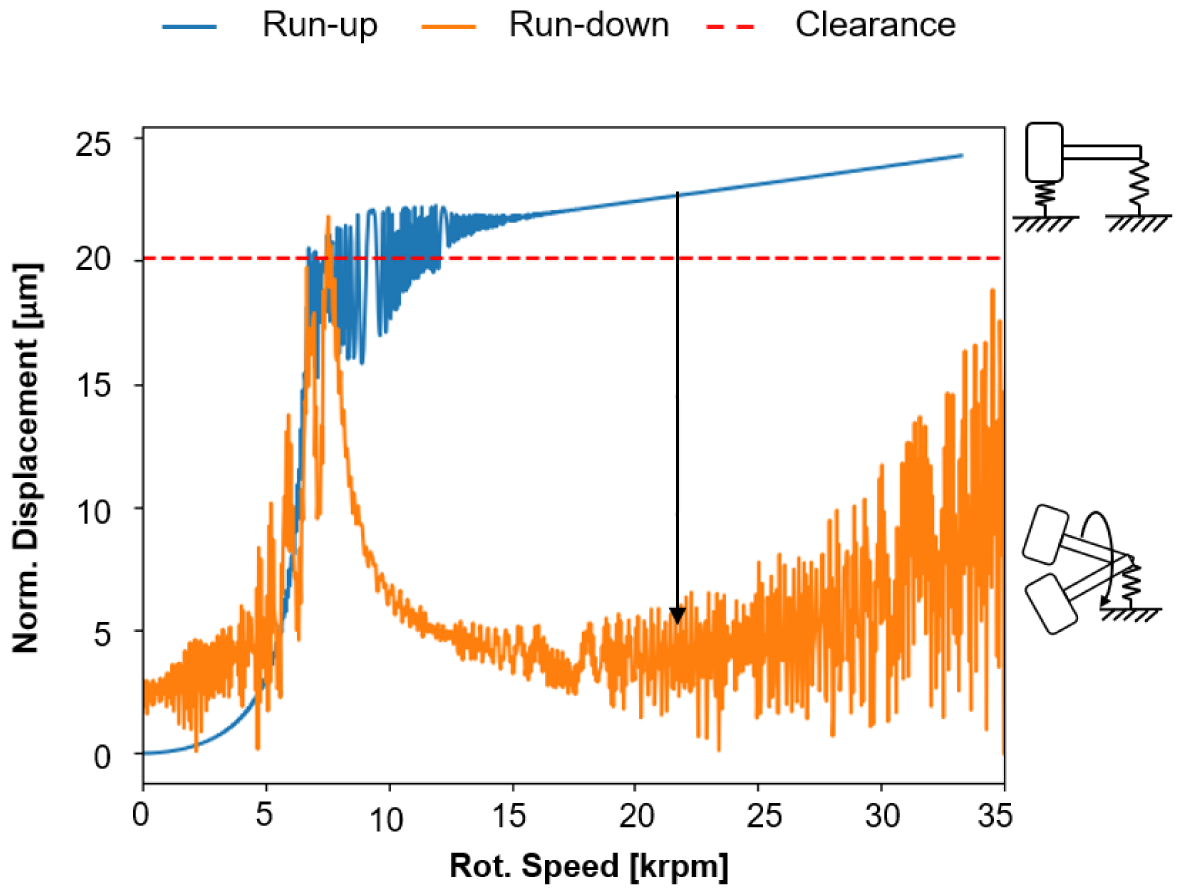
Supercritical operation of the cylindrical roller bearing (CRB).

Figure 15 shows the speed-dependent normalised radial displacement in idle mode for the cylindrical roller bearing for increasing (run-up) and decreasing speed (run-down) (see *Underlying data*). For the simulations of the operating behaviour of the cylindrical roller bearing, a radial bearing clearance of 37.5 *µ*m was assumed. For an increasing speed (run-up), the bearing goes through different operating conditions. First, the normalised radial displacement increases due to centrifugal force up to 5 krpm. At approximately 7.5 krpm, the normalised bearing clearance increases abruptly due to resonance and is almost used up. The unsteady radial displacement behaviour from 7.5 krpm up to about 15 krpm is due to an increasing skewing of the rollers, which stabilises from 15 krpm due to the increasing centrifugal forces.

**Figure 16.**
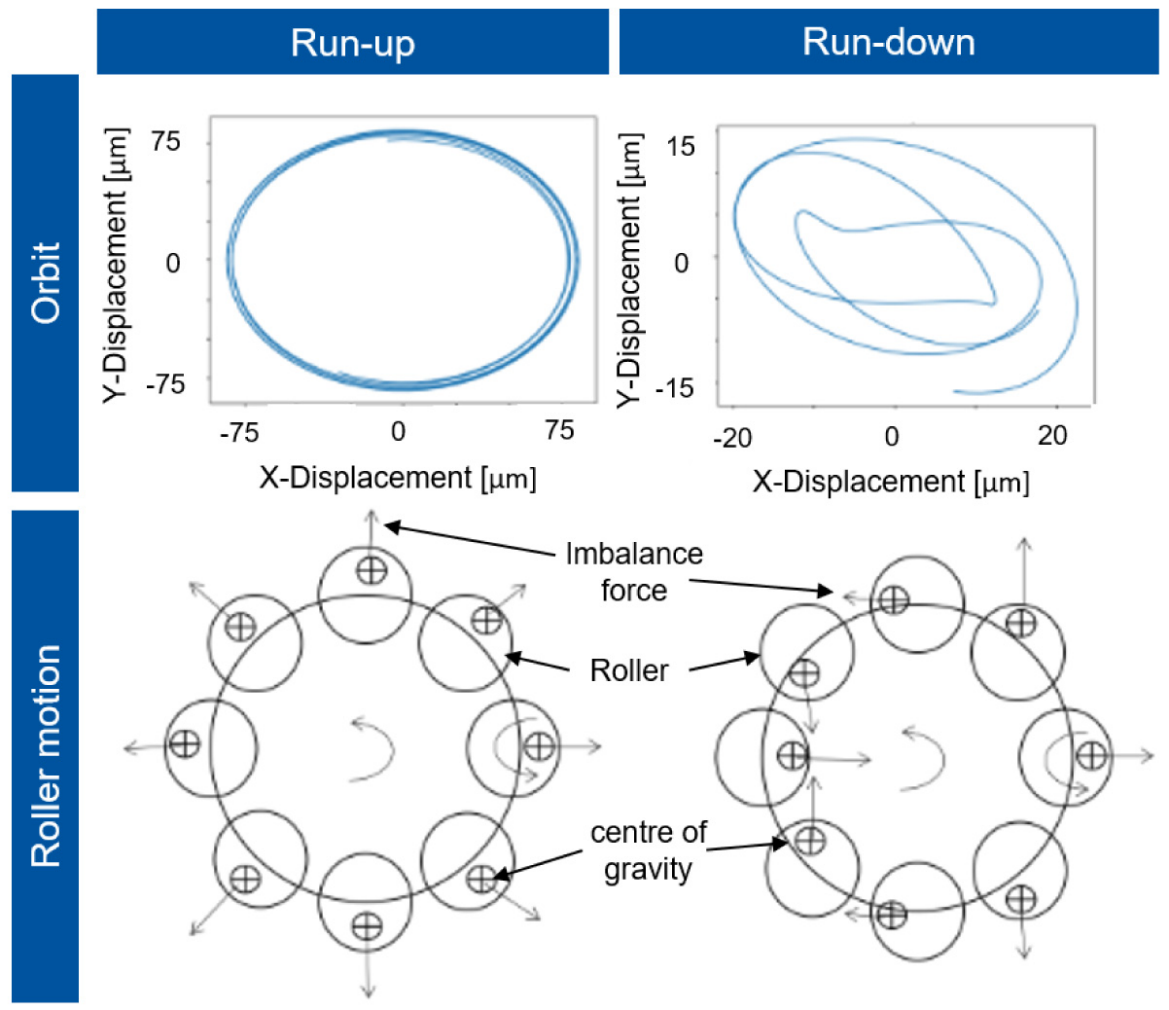
Shaft orbit and roller motion.

The imbalances of the single rollers are synchronous with the rotor whirl in this condition, so that the shaft rotates in a circular orbit to the rotor axis, which is shown in Figure 16. However, during deceleration (run-down), chaotic alignment of the rollers occurs due to bearing clearance and decreasing centrifugal force. The imbalances of the rollers are no longer synchronous with the rotor speed, so that a whirl with kinks forms and the response behaviour in the normalised displacement is significantly smaller.

For decreasing speed (run-down) starting from 35 krpm, the radial displacement is more unsteady, which results from the cantilevered state of the system in which the cylindrical roller bearing now behaves like a damper. At about 7 krpm, the bearing resonates with the rotor speed as the speed decreases, resulting in a peak of normalised displacement. Due to the non-uniform movement of the rollers and their contact conditions, skidding is expected in this operating condition. Determining the bearing friction due to these circumstances is much more complex. This critical state of the cylindrical roller bearing can be prevented by a compensation force through the radial force control, which leads to a lower excitation of the whole system. The calculations show that the operating behaviour of the cylindrical roller bearings has a major influence on the generator’s performance, especially in the high-speed range. On the one hand, the tests on the generator and on the single bearing test rigs can provide a better fundamental understanding of the effects acting on the bearing at high-speeds, e.g. roller skewing. On the other hand, radial force control can also improve the operating behaviour in the high-speed range by minimising friction in the cylindrical roller bearing. A better understanding of this theoretical phenomena will be established by both, the tests on the generator test rig, and the friction torque tests on the single bearing test rig.

## 5 Validation on a single bearing test rig

To validate the single bearing friction losses under the load and speed conditions as in the generator test rig, further investigations on a rolling bearing test rig with a bearing friction torque measurement system are necessary. In the following, the test rig and its measuring system for evaluating bearing friction is described. Furthermore, experimentally measured friction curves of the generator bearings are compared with friction calculation models.

### 5.1 Test rig set up

**Figure 17.**
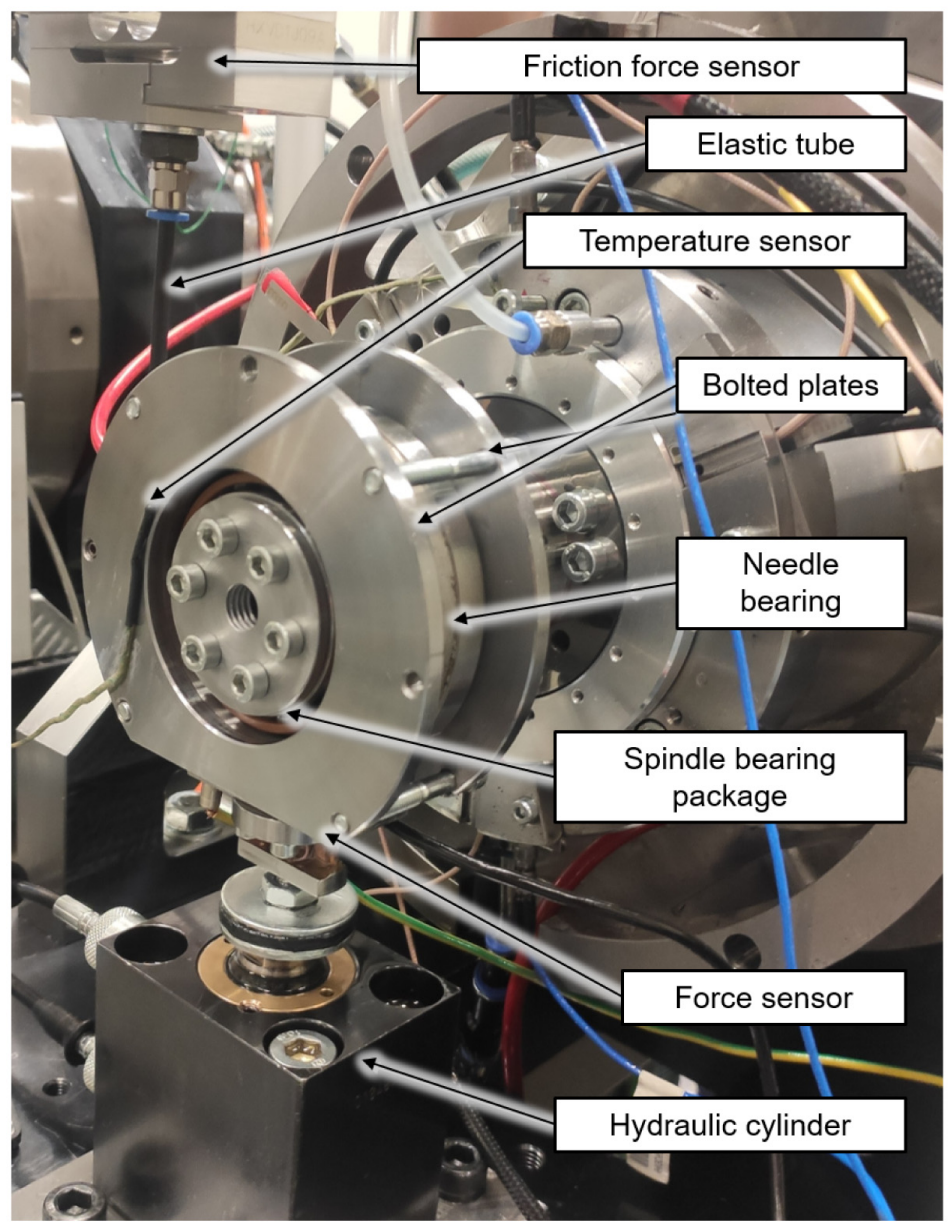
Radial load unit with friction measurement system.

For the investigations a modular test rig type is used, which is described in 15. In the case of the spindle and cylindrical roller bearings of size 7008 and N1008 from the generator test rig, the friction torque measurement is implemented by minor adjustments to the existing radial load unit. The test rig with friction torque measurement on the radial load unit is shown in Figure 17. The test bearings are a hybrid spindle bearing package positioned in a rigid O-arrangement in the load unit. The outer rings of the bearing package are preloaded with 65 N by two plates bolted to each other. The outer rings of the bearing package are rotatable supported by a needle bearing. During the test a high precision sensor of HBM type S2M, which is connected to the plates by an elastic tube, measures the bearing friction force of the radial load unit in range up to 20 N. The radial force is applied directly to the test bearings by a hydraulic cylinder and measured by a self made strain gauge-based sensor. In addition, the external bearing temperature of the load unit is measured.

### 5.2 Test program

**Figure 18.**
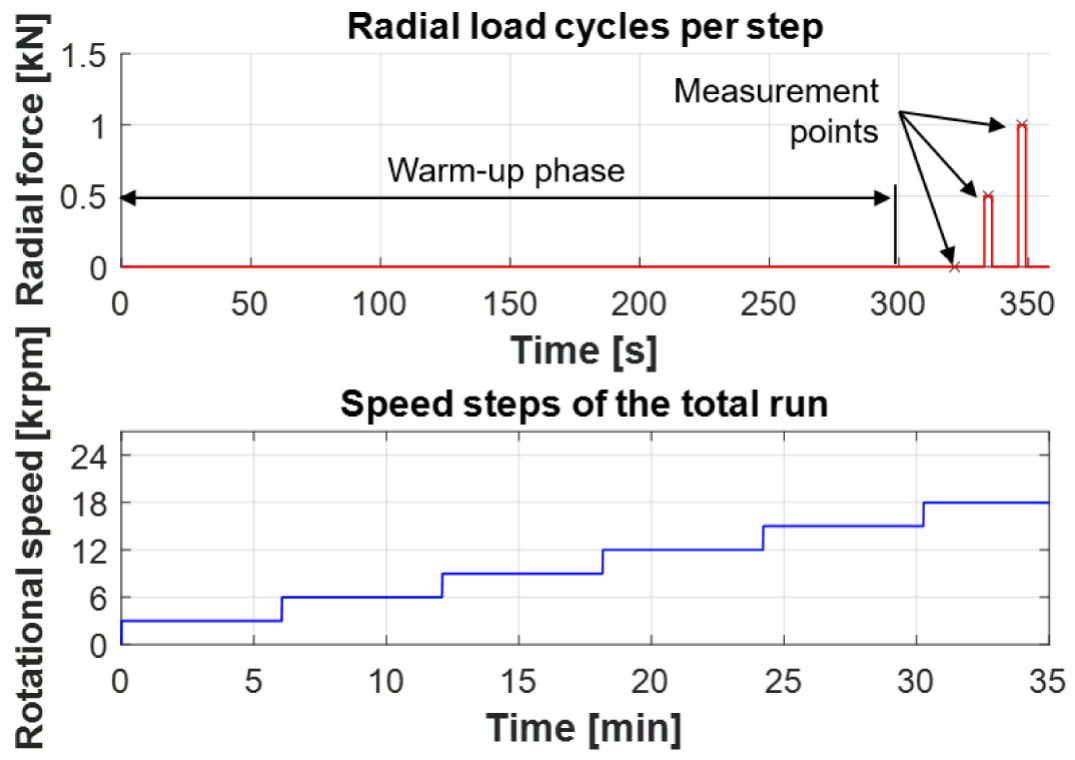
Test program.

The test program to measure the bearing friction is shown in Figure 18 (see *Underlying data* ^22^). The upper part of the figure shows the radial load for each speed step. After a warm-up phase of about 300 s, the bearing is subjected to short load phases with a radial force of 500 N and then 1,000 N. When the target force level is reached, the bearing friction is then determined as an average value, which is illustrated in the figure by the measurement points. This approach ensures that almost isothermal temperature level in the bearing. The lower part of Figure 18 shows the total speed step run. The bearing friction is evaluated in 3,000 rpm steps up to a maximum speed of 18,000 rpm. During the test, the bearings are run in an oil-air lubrication system with a fine-filtered spindle bearing oil of ISO VG viscosity class 68 and a lubrication flow rate of 150 *µ*l/h. In general, the test conditions are comparable to those in the generator test rig. 

### 5.3 Measured and calculated bearing friction

**Figure 19.**
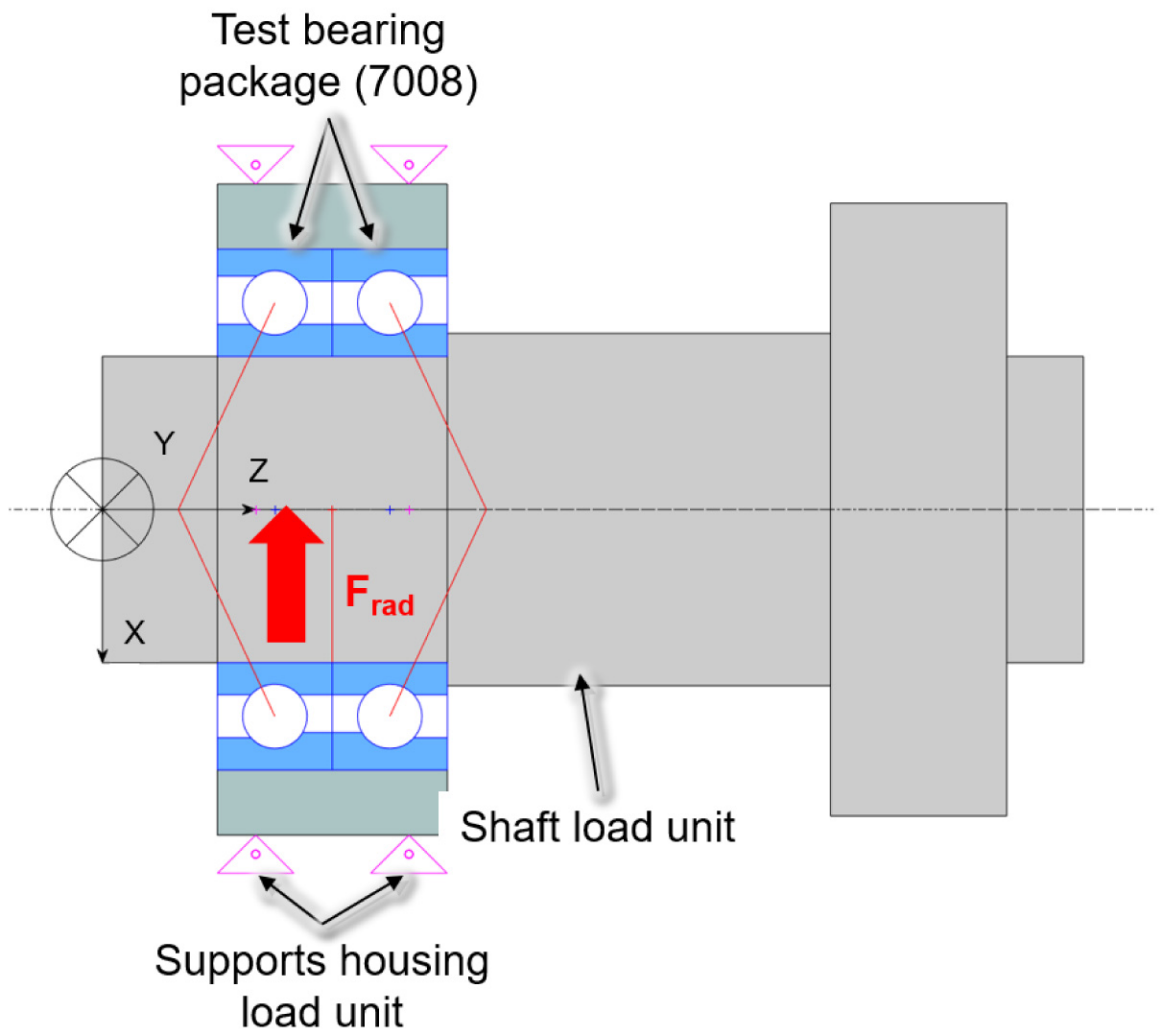
Simplified simulation model of the test rig.

To compare measurement and calculation, a simplified model of the test rig (see *Underlying data* ^22^) was built up in MTPlus (see *Software availability*), and is shown in Figure 19.

The underlying calculation of bearing friction is, as far as the author is aware, only implemented in the software. However, the procedure for this is described in ^18^. Since the focus of the investigations is on the spindle bearing package in the load unit and to shorten the calculation time, only the load unit is modeled. Since the load unit is supported only by the hydraulic cylinder when the force is applied radially, which is hard to model, and tilting of the shaft of the load unit relative to the bearings is negligible, the direction of the load is reversed in the model. The radial force is applied via the shaft, with the housing radially fixed by two supports. As a result, the force flow is reversed, but the absolute friction remains the same. The friction was calculated according to Steinert’s approach ^18^, taking into account the guide method of Tüllmann with varying lubricant film height ^16^. According to Steinert the friction components are divided in the irreversible deformation work $M_{A\sum }$ , the the rolling work $M_{B\sum }$ , the bore friction $M_{C\sum }$ between the bearing rings and ball, the sliding friction between bearing rings and cage $M_{D\sum }$ as well as balls and cage $M_{E\sum }$ . By calculating and summing the single shares, the total friction *M _tot_* in the bearing is determined using eq. 8.


$$\;M_{tot}=M_{A\sum }+M_{B\sum }+M_{C\sum }+M_{D\sum }M_{E\sum }\;(8)$$

**Figure 20.**
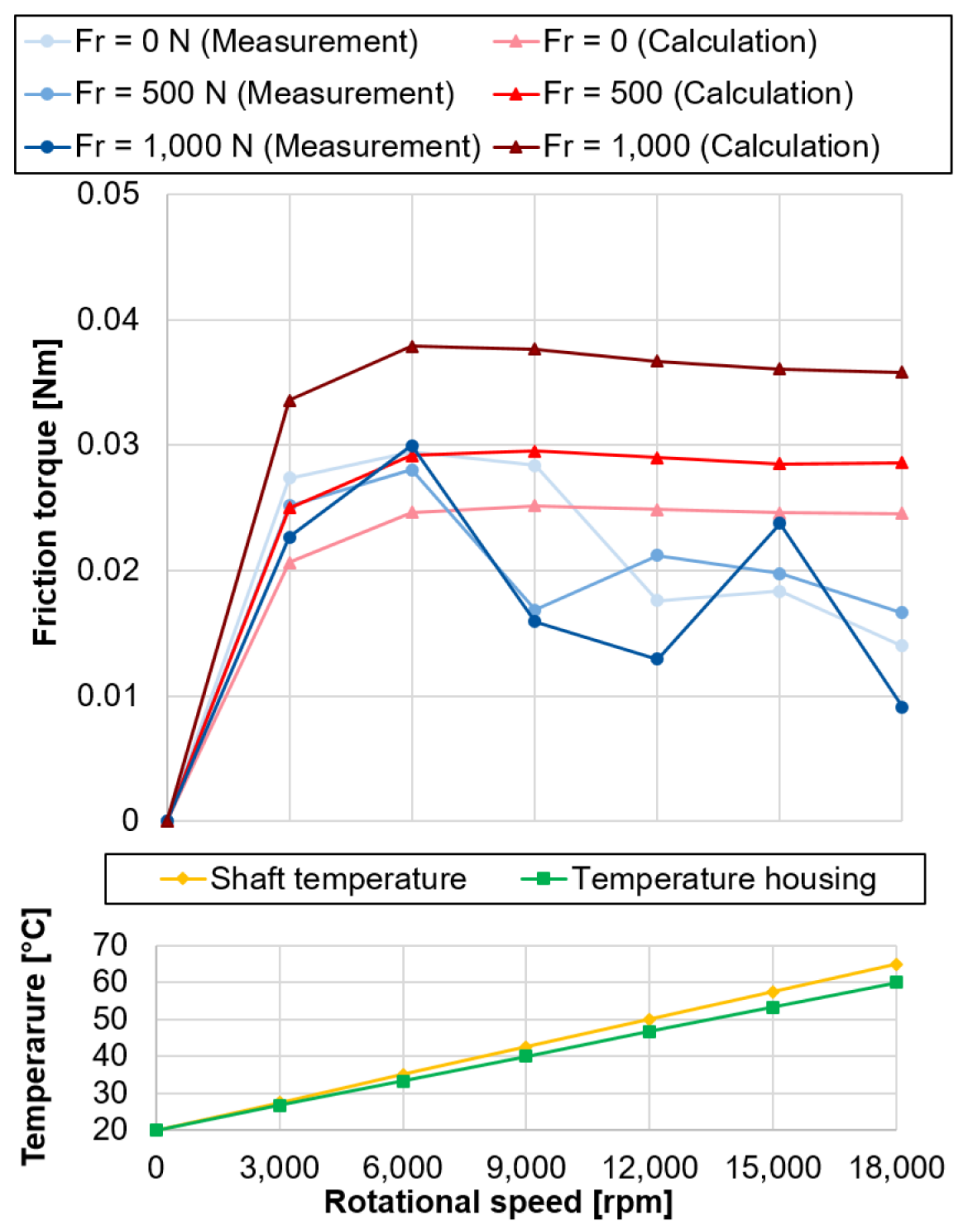
Comparison of measured and calculated bearing friction with temperature profiles. FR, radial load.

The comparison between measured and calculated bearing friction with the assumend temperatures for the calculation is shown in Figure 20 (see *Underlying data* ^22^).

The calculated bearing friction losses represent the summarised maximum friction for the outer and inner ring. The comparison between measurement and calculation shows that they are in the same order of magnitude and provide good comparability at higher speeds above 15,000 rpm. Nevertheless, the results differ in the low speed range, the measured bearing friction increases significantly at first, reaches its maximum value at 6,000 rpm and then drops again with increasing speed, before the measured values increase again from 15,000 rpm. A possible explanation for the deviating behaviour is the strong influence of the temperature on the rigid arrangement of the test bearings. As the speed increases, the difference in temperature between the outer and inner ring increases, so that the preload is successively reduced, which also results in a drop in friction. From 15,000 rpm the kinematic friction component increases more strongly, which in turn is reflected in the increase in measured bearing friction. Furthermore, with increasing radial load, there is a redistribution of the load in the bearing. One half of the rolling element set is more heavily loaded and the other less heavily loaded, which is also reflected accordingly in the friction contact surfaces. The two mechanisms must be taken into account in the measured friction, as they cancel each other out, so that almost no influence of the radial load on the bearing friction can be determined.

## 6 Conclusions and outlook

Radial force control represents an important technology for increasing the performance of aircraft generators and their bearings. Previous studies have already shown the versatility of this technology for motors and generators. For example, shaft imbalances at speeds of up to 3,000 rpm can be suppressed. However, the influence of externally applied radial forces has not yet been investigated for radial force control. This article therefore presents the setup and design for investigating these influencing factors. The calculations for different operating conditions show that the bearing loads and friction can be reduced with radial force control, so that the lifetime of the bearings can be increased. In particular, the operation of the cylindrical roller bearing in the generator with bearing clearance under high speeds and additional radial load shows that friction-related dynamic effects can also be expected in the bearing, which must be investigated experimentally. The additional validation of the operational conditions of the bearings in the generator by tests on a single-bearing test rig with friction torque measurement allows to analyse current friction calculation approaches on the basis of the case study of radial force control and to extend them if necessary. Besides that, the knowledge gained in the project can also be transferred to other generator types. Once the bearing monitoring system and radial force control are successfully tested, they can also be transferred to other high-speed shaft-bearing systems, such as in the tool spindle, and tested for loads with higher amplitudes and frequencies. Furthermore, in the course of this project, measuring methods for the determination of bearing friction are developed further and calculation methods for the characterisation of operating conditions in shaft-bearing systems are optimised. The interdisciplinary cooperation between researchers from the fields of electrical engineering, measurement and test bench technology and simulation technology creates synergies and a transfer of knowledge.

## Data availability

### Underlying data

Zenodo: Design of an aircraft generator with radial force control. https://doi.org/10.5281/zenodo.6464967 ^22^.

This project contains the following underlying data:

- Figure 7 - Radial shaft displacement (bearingless mode).csv
- Figure 9 - MTPlus model of the generator test rig.csv
- Figure 10 - Variation of the generator controlled radial force.csv
- Figure 11 - Maximum contact pressure on the inner ring.csv
- Figure 12 - Lifetime calculation of the inside positioned.csv
- Figure 13 - Max. friction in the spindle bearings.csv
- Figure 18 - Test program.csv
- Figure 19 - Simplified simulation model of the test rig.csv
- Figure 20 - Comparison of measured and calculated bearing.csv

Data are available under the terms of the Creative Commons Attribution 4.0 International license (CC-BY 4.0).

The data for the transient calculation of the cylindrical roller bearing in Figure 15 cannot be provided in raw form due to the large amount of data. Readers can request access to the data by contacting the author Mr. Barry James: barry.james@hexagon.com.

## Software availability

The source code of the bearing calculation software MTPlus and Nexus were previously developed by several contributors at the Laboratory for Machine Tools and Production Engineering (WZL) of RWTH Aachen University, and not by the authors of this article. The source code cannot be provided due to confidentiality and competitive reasons since this software can be purchased commercially. Interested readers can contact Mr. Stephan Neus ( s.neus@wzl.rwthaachen.de) to request information on MTPlus, and Mr. Maik Hoppert ( maik.hoppert@hexagon.com) to request information on Nexus, however full access to the software code cannot be granted. Nevertheless, the authors confirm that sufficient instruction and underlying data has been provided in this article to enable replication of the proposed method by readers.

## Ethics and consent

Ethical approval and consent were not required.
