# Supplementary material for: Design of an aircraft generator with radial force control
Source: Open Res Eur. 2023 Apr 17;2:73. Originally published 2022 Jun 6. [Version 3] doi: 10.12688/openreseurope.14684.3 (PMC10445881; doi:10.12688/openreseurope.14684.3)
Supplement: Peer review history

# Peer review: Reviewer response for version 3

**DOI:** 10.21956/openreseurope.17119.r31176

**Published:** 2023-04-27

**Authors:** Rubén Lostado Lorza

**Affiliations:** 1 Department of Mechanical Engineering, University of La Rioja, La Rioja, Spain

Once the corrected work has been analyzed again and seeing that its authors have improved it according to the suggestions proposed, from my point of view I can only affirm that the work could be definitively indexed.

 With my best wishes

 Rubén.

Is the rationale for developing the new method (or application) clearly explained?

Yes

Is the description of the method technically sound?

Yes

Are the conclusions about the method and its performance adequately supported by the findings presented in the article?

Yes

If any results are presented, are all the source data underlying the results available to ensure full reproducibility?

Yes

Are sufficient details provided to allow replication of the method development and its use by others?

Partly

Reviewer Expertise:

Mechanical Engineering; Finite Element Analysis; Bearings.

I confirm that I have read this submission and believe that I have an appropriate level of expertise to confirm that it is of an acceptable scientific standard.

# Peer review: Reviewer response for version 2

**DOI:** 10.21956/openreseurope.16333.r30966

**Published:** 2023-03-28

**Authors:** Rubén Lostado Lorza

**Affiliations:** 1 Department of Mechanical Engineering, University of La Rioja, La Rioja, Spain

The manuscript is very well explained and justified, and shows the design of an aircraft generator with radial force control. However, the work has some points that should be justified to clarify the reader.

 In this paragraph "The radial force is applied directly to the test bearings by a hydraulic cylinder and measured by a self made strain gauge-based sensor". The authors of the paper should explain more in depth what they mean by the self made strain gauge-based sensor.

 It would be interesting to include a table in which the % of maximum error for various rotation speeds between the data obtained experimentally and those obtained by simulation are attached, which are presented in figure 20.

 The authors of the research work propose an equation (6 and 7) in which they take into consideration the stiffness of the spindle bearing package cSBP and the stiffness of the cylindrical roller bearing cCRB. This stiffness obviously depends on the degree of preload, load and clearance of the bearings. Do the authors know the values of this stiffness based on these parameters?

Is the rationale for developing the new method (or application) clearly explained?

Yes

Is the description of the method technically sound?

Yes

Are the conclusions about the method and its performance adequately supported by the findings presented in the article?

Yes

If any results are presented, are all the source data underlying the results available to ensure full reproducibility?

Yes

Are sufficient details provided to allow replication of the method development and its use by others?

Partly

Reviewer Expertise:

Mechanical Engineering; Finite Element Analysis; Bearings.

I confirm that I have read this submission and believe that I have an appropriate level of expertise to confirm that it is of an acceptable scientific standard, however I have significant reservations, as outlined above.

# Response

**Published:** 2023-03-30

**Authors:** Marcus Gärtner

Dear Prof. Lostando Lorza, Thank you very much for your good and constructive feedback. We have revised our manuscript with regard to your comments and would like to respond briefly. Further information on the radial force sensor has been added to the relevant passage, and a source with further details has also been added. To improve the comparison between measured and calculated friction values, a table was prepared with the percentage deviation. Furthermore a table was added in section 4 giving the missing bearing stiffnesses for a discrete operational point oft he generator bearings. We hope to have clarified your open points and remain with kind regards

# Peer review: Reviewer response for version 2

**DOI:** 10.21956/openreseurope.16333.r30171

**Published:** 2022-10-14

**Authors:** Steffen Ihlenfeldt

**Affiliations:** 1 Institute of Mechatronic Engineering, Dresden University of Technology, Dresden, Germany

The authors have considered my comments in the revised manuscript. All my questions have been commented plausible. In my point of view, a high quality of the article has achieved.

 I do not have further comments.

Is the rationale for developing the new method (or application) clearly explained?

Yes

Is the description of the method technically sound?

Partly

Are the conclusions about the method and its performance adequately supported by the findings presented in the article?

Yes

If any results are presented, are all the source data underlying the results available to ensure full reproducibility?

No

Are sufficient details provided to allow replication of the method development and its use by others?

Yes

Reviewer Expertise:

machine tools, production systems, adaptive control systems and cognitive production processes

I confirm that I have read this submission and believe that I have an appropriate level of expertise to confirm that it is of an acceptable scientific standard.

# Peer review: Reviewer response for version 1

**DOI:** 10.21956/openreseurope.15861.r29500

**Published:** 2022-07-13

**Authors:** Steffen Ihlenfeldt

**Affiliations:** 1 Institute of Mechatronic Engineering, Dresden University of Technology, Dresden, Germany

Radial force control represents an essential technology for increasing the performance of aircraft generators and their bearings. The paper presents the setup and design steps of high-speed generators with radial force control. The objective is to reduce bearing friction and compensate for disturbing forces on the bearing points using a radial force generated by the generator’s magnetic field. For this purpose, a control concept has been developed and investigated.

 Primarily, the paper deals with constructing a test rig for radial force control and presenting a possible control concept. The focus lies on investigations and simulations regarding the rolling bearing behavior of the rotor bearing. The investigation of the actual control strategy is not part of the paper.

 The starting point of the investigations is well presented, and relevant papers on the classification of the problem are included.

 The bearing arrangement at the force application points in Figure 2 is not clearly visible. It apparently exists of angular contact ball bearings in an O-arrangement. The two sides appear to be preloaded. How was this arrangement prevented from transmitting forces to the test rig, especially when the shaft is bent? Do additional forces or moments arise at the bearing points of the force application due to tilting influencing the measurement?

 A comprehensive measurement setup with acceleration sensors, displacement measurement systems, and force measurement systems enables the dynamic behavior of the generator system to be investigated and the radial force control to be qualified. The investigations on reducing the frictional torque were carried out on a separate test rig.

 The described procedure for calibrating the generator shaft is comprehensible. The calculation also considered the relevant elasticity of the components in the force flow.

 In the bearing-shaft simulation model, the bearing of the load unit is not considered. The O-arrangement of the bearing has a high tilting rigidity. Can the resulting tilting moments during shaft bending influence the investigations?

 In the simulation without the rotor bearing, these effects seem to have been considered. How were they taken into account in the control model?

 Figure 7 should illustrate that this simulation took place at 5,000 rpm.

 The used simulation programs are comprehensibly documented in other publications.

 All used methods were described or were cited with sources accordingly. The programs were disclosed, and open source alternatives were also shown. Only specific measurement series for bearing characterization were not published due to obligations of confidentiality.

 All essential measurement results for evaluating the rolling bearing behavior are available. Measurement series for evaluating the control behavior of the radial force control were not part of the paper; therefore, they are not available. Especially for the calibration of the trainer, a comparison of simulation results and trainer measurement results would be desirable to evaluate the calibration method.

 The simulations on rolling bearing behavior and the behavior of the spindle bearing-shaft system support the thesis that radial force control can contribute to minimizing bearing friction and increased service life.

Is the rationale for developing the new method (or application) clearly explained?

Yes

Is the description of the method technically sound?

Partly

Are the conclusions about the method and its performance adequately supported by the findings presented in the article?

Yes

If any results are presented, are all the source data underlying the results available to ensure full reproducibility?

No

Are sufficient details provided to allow replication of the method development and its use by others?

Yes

Reviewer Expertise:

machine tools, production systems, adaptive control systems and cognitive production processes

I confirm that I have read this submission and believe that I have an appropriate level of expertise to confirm that it is of an acceptable scientific standard, however I have significant reservations, as outlined above.

# Response

**Published:** 2022-09-09

**Authors:** Marcus Gärtner

Dear Prof. Ihlenfeldt thank you very much for your review and your helpful and positive feedback. We have implemented your comments on Figures 3 and 7 and uploaded a new version. In the following, we would like to respond to your questions.

*“How was this arrangement prevented from transmitting forces to the test rig, especially when the shaft is bent?”*

 In general, the arrangement must be supported by the housing, which is stiffly clamped by the two feet and the covers. The cylindrical roller bearing with clearance provides radial force control with an additional degree of freedom to control the rotor position. Therefore, most of the applied interference force is absorbed by the spindle bearings and the radial force control of the generator itself. A comparatively smaller part of the interference forces is absorbed by the cylindrical roller bearing.

*“Do additional forces or moments arise at the bearing points of the force application due to tilting influencing the measurement?”*

 The spindle bearings are selected with a specific preload class, which is set very precisely by the manufacturer. Due to the defined fixation of the bearings with a shaft nut with a defined torque, no additional forces are expected from the bearing itself apart from its own weight and the centrifugal forces at high speeds. Nevertheless thermal forces can arise in an endurance run, which can be compensated for by the axial movability of the floating bearing. Generally, shaft bending due to the interference forces is expected and even desired, to estimate and compensate the applied forces by radial force control. The calculations show, that the expected displacements lie in a range that the eddy current sensors can detect. How large these displacements actually are must be evaluated in the tests on site.

*“In the bearing-shaft simulation model, the bearing of the load unit is not considered. The O-arrangement of the bearing has a high tilting rigidity. Can the resulting tilting moments during shaft bending influence the investigations?”*

 Depending on the configuration, the shaft is supported in different ways by the bearings, which is the reason for the different considerations of the load units.

**Generator with bearings:**

 The load unit is deliberately not taken into account in the simulations, as an accurate representation of the displacement and stiffness at the support points is extremely complex and can only be sensibly implemented with tests on the test bench. It is therefore assumed, that the deformation of the shaft is significantly greater than the deformation of the load unit with bearings, so that exactly the force is transmitted to the support points that is also generated by the load unit.

**Generator without bearings:**

 The complete load unit is taken into account, as it is supported on both sides by four sensors. In this case, it can be assumed that the positions of the load units are fixed and their stiffness can be approximated by the sensor data, which have the lowest stiffness compared to the housing and the bearings. Additional tilting moments can certainly occur in the tests, which can also influence the measurements. However, their influence is not considered to be too great, as relatively small radial loads of up to 500 N per cylinder are applied. The measurements must show how great their influence actually is.

*“In the simulation without the rotor bearing, these effects seem to have been considered. How were they taken into account in the control model?”*

 By means of the simulations, a preliminary design of the control model is carried out. However, the parameters of the control model must be continuously adjusted and optimised by the calibration procedure and the measurements in generator mode. In particular, the vibration behaviour of the tilted load units in generator mode and their influence on the rotor dynamics can only be accurately determined by the tests, which will certainly require even more extensive adjustments to the control loop.
